# Editorial

**Published:** 1897-03

**Authors:** 


### Editorial.

We publish elsewhere in this number an amusing letter, taken from an exchange. The writer very wittily takes novel writers to task for their absurd conception and depiction of disease, and their equally impossible medical men.

His humorous criticism applies very well to the novel of a decade ago, but it breaks down before the realistic fiction of to-day. The novelists have stopped talking through their hats. They go to the proper sources for medical information, and give it to their readers fairly correctly. The Sunday newspapers have cleared away the mist that obscured the subject of disease to the lay reader, and have made him a hard man to fool.

It is no longer expedient for the villain to drop dead at the height of some dramatic situation from an unexpected cardiac lesion, nor does the heroine fall a victim to acute tuberculosis as a result of unrequited affection. The blasé public demands something different. However, Hon. Capt. Bilgewater still drags out a tedious convalescence from an Indian fever on the Riviera, where he meets fate in the form of the young Lady Featherstonehaugh, beautiful but fragile, and recovers health and vigor under the fostering care of Cupid, M. D.

This variety of ailment is distinguished by a conservatism which reduces the chance of erroneous description to a minimum. Jungle fever requires no particularization of symptoms, and is thoroughly safe ground—for the writer, if not for the patient. It makes no claim to the hectic flush and the hacking cough, but demands only the glittering generalities of,an interesting pallor, attractive languor and a slight transparency of the large, white hand.

Non-committal diseases like this are likely to persist throughout the ages, among writers of the Laura Jean Libbey cult. But there are bolder spirits who would depict the social evil "with all the brutal justice of a photograph," and disdain to concern themselves with the dull but highly respectable maladies of the fiction characters of the past. *Nihil humanum a me alienum puto* is the principle behind which modern realists seek justification for their creations. Hence syphilis and sexual perversion have pushed out the fractured vertebrae and the tuberculous lung of other days.

While the type of disease has been changed to suit the rigidly realistic requirements of *fin de siecle* fiction, sterility of the novel heroine is still common. The French are particularly fond of inflicting the not unmitigated curse of barrenness upon their female characters in fiction for, were it otherwise, Bel Ami, Mensonges, Sappho and Mlle. de Maupin would lose the charm of their Gallic naughtiness and such poisonous fungi as O'Monroy, Zanrof, and Catulle Mendes, would have to retire permanently from business.

There is no morbid anatomy in this convenient lack of fertility, unless it be accidental. A worldly wisdom in the matter of prevention of disagreeable consequences of moral lapses by mechanical appliances offers the explanation.

The ever-fickle public is already becoming restless and dissatisfied with this pathological era and is tired of having itself dissected and analyzed. It wants to be amused, not chidden and criticised. There are not lacking signs that their desires are to be gratified. A clean, wholesome romance promises to replace the morbid productions of the realistics. With such a wholesome change, we must expect more broken backs and gunshot injuries and surprising recoveries, and preternaturally wise doctors, but we will be well content, for it will mean that the stench of degeneracy has been removed. Even a return to the frank obscenity of Rabelais and the Decameron would be an agreeable change from the festering nastiness of the sophisticated novel of to-day. And that would represent the extreme backward swing of the pendulum, which we have no reason to expect or fear. May the type of Doyle, Weyman, Crane, and Crockett persist, and the pages of the novel to come be as full of begetting as the Chronicles of the Old Testament.

---

The Medical Association of Georgia will meet in Macon, April 21-24. An interesting program is promised and it is hoped that there will be a full attendance. Dr. Howard J. Williams, chairman of the local committee of arrangements is exerting himself to make the meeting a pleasant one from a social standpoint, and the profession in Macon are lending him their aid. The few days of rest can be profitably and agreeably spent and all are urged to come.

---

The Atlanta Medical Society will hereafter hold its meetings in its new quarters in the Equitable building. Dr. Noble, the president, is creating a revival of interest in the society and expects every member to do his duty. The January banquet was a distinct success and doubtless inaugurated a renewal of prosperity in the affairs of the society. There is no valid reason why interest in the society has languished of late years. It is essential to an interchange of thought and opinion among active practitioners; without it, the life of the physician is one of stagnation. A home of its own will do much toward a re-establishment of interest and will tend to bring out the members who habitually absented themselves on account of dissatisfaction with the former place of meeting. Dr. Noble deserves the congratulations of the society for his telling efforts in its behalf.

